# Alternative Organizations as Systems Hijacking: The Commercial Trust as a Thought Experiment

**DOI:** 10.1177/00076503221084647

**Published:** 2022-04-03

**Authors:** Heather M. Hachigian

**Affiliations:** 1 Royal Roads University, Victoria, British Columbia, Canada

**Keywords:** alternative organizations, community investment community wealth, governance, thought experiment

## Abstract

The COVID-19 crisis has renewed interest in alternative forms of organizing business and investment but our understanding of how these organizations can transform social systems is limited. The purpose of this article is to contribute to this understanding. In the context of one of the greatest transfers of wealth in global retail history that could see unprecedented numbers of businesses close or sold to distant, private interests, the article performs a thought experiment using the analogy of a commercial trust to encourage new ideas and critical reflection on community wealth building. The article introduces systems hijacking—a process of leveraging incumbent forms and systems in which they are embedded for new purposes—as an analytically useful concept for understanding how alternative organizations can transform social systems. The article finds organizational governance is necessary to transcend structural deficiencies in inherited or borrowed forms to make way for transformation.

The COVID-19 crisis is exacerbating one of the greatest transfers of wealth in global retail history. Small businesses—many already at risk of closing or being sold to distant investors on unfavorable terms—are facing accelerated succession timelines due to pandemic-era policies that privilege larger corporations (Fairlie, 2020; Project Equity, 2020). Small businesses are critical to community wealth by providing local employment, spending and investment, and the potential loss of small businesses from communities across North America is generating renewed interest among practitioners and policymakers in alternative ownership models to ensure these wealth-generating assets remain in communities (e.g., Democracy Collaborative, Project Equity). Organizational scholarship is also paying increasing attention to alternative ways of organizing business and investment in pursuit of social and community goals (Mair & Rathert, 2021). But scholarship has largely neglected the efficacy of alternative organizations in affecting and transforming institutional arrangements and social systems in which these organizations and the problems they address are embedded (de Bakker et al., 2020; Mair, 2020; Mair & Seelos, 2021; Mair, Wolf, & Seelos, 2016; Spicer, 2020; Stephan et al., 2016; Vestergaard et al., 2020). This article addresses this lack of understanding and asks what are the mechanisms and conditions for alternative organizations working within market-based systems to transform the social systems that underpin community wealth erosion.

Achieving this objective requires engaging with issues of power of alternative organizations to compete with private investors and preserve ownership of wealth-generating assets in communities. This article identifies three key criteria for an alternative organization to compete with private interests: scalability, durability, and efficient representation of community interests. A review of existing models for community wealth building in the Anglo American context shows a lack of models designed to leverage the power necessary to meaningfully compete with private investors. Given the lack of existing models in the Anglo American context that serve this purpose, the article conducts a thought experiment (Kornberger & Mantere, 2020) to imagine a scenario that would allow communities to acquire and operate businesses at a scale that has the potential to transform the social systems that hold the problem of community wealth erosion in place. Social systems are “causal architectures that are shaped by people, their beliefs and ambitions, their skills and access to resources, and the norms and rules by which they relate to each other and their environment” (Seelos & Mair, 2018, p. 36). The thought experiment uses the analogy of the commercial trust, which has long been used by private interests to build and protect wealth (Langbein, 1997; Pistor, 2019). In our model, the investment entity owes fiduciary duties to community and is governed by articles that establish its purpose.

At the heart of this inquiry is a paradox—documented elsewhere in the literature on investment organizations—that relates to using inherited structures for new purposes. On one hand, investment organizations with fiduciary duties look to convention—and not innovation—to guide their decision-making (Clark, 2011; Clark & Monk, 2017). On the other hand, investment organizations that prioritize democratic representation by leveraging innovative structures are vulnerable to political interference (Clark & Monk, 2010, 2017) and conflicts between community members (Haugh, 2021) that can undermine financial performance and, ultimately, the efficacy of the organization to achieve its social or community objectives.

The article advances scholarship on alternative organizations in the following ways. First, the article addresses an important gap in the literature by interrogating the form, function, and governance of capital providers in alternative organizing to understand the impact they have on their investees. In doing so, the article “bring[s] renewed attention to power as a crucial factor in theorizing about alternative organizations” (Mair, 2020, p. 352). Indeed, the growth of alternative investment organizations—from impact funds, community development financial institutions, and social venture capital firms, among others—offers promising potential for the future of businesses with social and community purpose. But alternative organization scholarship has paid little attention to organizations that perform investment functions and their impacts on the entities in which they invest (Lall, 2019; Mair, 2020). The COVID-19 crisis has brought into stark relief the relative power of different actors (Baines & Hager, 2021), and it is feasible to imagine a more strategic response from community sector organizations that engages with issues of power (see Spicer, 2020). For example, this article is inspired by work with a community partner organization to explore new mechanisms to address the problem of wealth erosion in our community.

Second, findings from our thought experiment reveal the potential and limits of using existing forms and the institutional context in which they are embedded to achieve new purposes. This article finds that organizational governance can offer a temporary solution to the dilemma of using inherited or borrowed forms for new purposes, and there is value in studying complementarities between various theories of transformation at multiple levels of analysis (e.g., individual, organizational, field). For example, using inherited forms for new purposes could prevent further erosion of community wealth until innovative forms that are fit-for-purpose are legitimized and widely adopted. In this way, exposing efforts of alternative organizations to address complex social and environmental problems allows us to understand the role of organizational governance not only for navigating an alternative organization’s institutional plurality (Mair, Mayer, & Lutz, 2015) but also for achieving their external goals related to affecting and transforming social systems.

Third, the article demonstrates the value of thought experiments to organizational scholarship. Thought experiments are useful when empirical data are nonexistent, and for facilitating critical reflection on existing approaches and surfacing new questions for empirical investigation (Kornberger & Mantere, 2020). In the alternative organization scholarship, this methodological approach is needed to imagine new ways for organizing business and investment that extend beyond the current focus on social enterprises and their hybridity (Battilana & Lee, 2014) and to imagine what could be in relation to addressing complex social and environmental challenges. In doing so, the article engages in an analytical process of retroduction (Sayer, 1992), where the direction of analysis flows from the effects to their causes to answer what would need to be true to generate desired outcomes.

The article is structured as follows. First, the article introduces the concept of systems hijacking—a process of leveraging existing structures and the institutional arrangements in which they are embedded for new purpose—as an analytically useful concept for understanding how alternative organizations can transform social systems that cause the problems they are seeking to address. Second, the article introduces the problem of community wealth erosion, substantiates the need for a thought experiment, and explains the relevance of the methodology to organizational scholarship. A thought experiment is used to identify the limits and unintended consequences of hijacking the commercial trust structure for community wealth building. The discussion elaborates on how organizational governance can transcend the paradox of using inherited structures for new purposes and the limitations of this approach. The article concludes with a reflection on its limitations and identifies new research avenues for advancing our understanding of the role of alternative organizations in transforming social systems.

## Theoretical Motivations

Recent scholarship on alternative ways of organizing for social purpose calls for a shift away from the dominant focus on the hybridity of social enterprise to consider how a broad range of alternative organizations could contribute to tackling complex social and environmental issues (Bansal et al., 2021; DeJordy et al., 2020; Dorado & Ventresca, 2013; Mair & Rathert, 2020; Mair & Seelos, 2021). This article builds on the work of scholars who emphasize the analytical value of focusing on the organization and its activities for affecting and transforming institutional arrangements underpinning systemic social problems (Mair, Wolf, & Seelos, 2016; Seelos & Mair, 2014, 2018). Within this literature, scholars have described and explained the mechanisms underpinning different approaches that alternative organizations take to affecting and transforming their institutional contexts in which problems they aim to address are embedded. For example, Mair, Wolf, and Seelos (2016) describe how a social enterprise uses scaffolding—a process that enables and organizes transformation of behavior and interaction patterns—to mobilize institutional, social, organizational, and economic resources, stabilize new patterns of interaction along an alternative social order, and conceal the pursuit of undesirable goals.^1^

This article introduces *systems hijacking* as another potential archetype for understanding how alternative organizations engage in the work of transforming the causal architecture underpinning community wealth erosion. Systems hijacking refers to the process of appropriating the structures and institutional arrangements in which they are embedded for an alternative purpose, and in doing so, transforming the system. In our context, the social system refers to the norms, rules, beliefs (institutional arrangements), and access to resources that shape how different actors within the capitalist system build and protect their wealth and that give rise to the social problem of community wealth erosion. Capitalism is defined for the purposes of this article as a process of minting assets by assigning to them the legal attributes of convertibility, durability, universality, and transferability (Pistor, 2019). This definition positions inequality as an inherent property of capitalism; incumbent wealth holders and their lawyers use indeterminate and malleable qualities of the law to protect and grow their assets over time (Pistor, 2019). To be sure, existing theories could be used to explain how alternative organizations leverage existing structures to transform their institutional contexts and address systems that lock in social problems they are seeking to solve (see Table 1). Perhaps the closest to the archetype of systems hijacking is the process of institutional conversion, where political actors redirect institutions toward purposes beyond their original intent (Streeck & Thelen, 2005; Thelen, 2004). Like systems hijacking, institutional conversion is enabled by ambiguity in existing institutions (Hacker et al., 2015). But there are also important differences, detailed in the section below. Indeed, there is no shortage of theories on how to change institutions and systems underpinning economic and social life (see Speth & Courrier, 2020), and it is beyond the scope of this article to offer an exhaustive review or analysis of these alternatives. The purpose of the following discussion is to identify three key features of systems hijacking that justify the introduction of a new archetype.

**Table 1.** Comparing Systems Change Archetypes.

Systems hange archetypes	Level of analysis	Sequence of change	Mechanisms used by disenfranchised for change
Transformation Fraser (2020)	Institutional	Function follows form	Dismantle root causes and rebuild institutions along socialist lines
Non-reform reforms Gorz (1967)	Institutional	Function follows form	Progressive appropriation of power by working against the prevailing system
Institutional conversion Thelen (2004)	Institutional	Unspecified (ambiguity may sustain form long after function changes)	Power through access to political institutions to re-interpret ambiguous rules
Field encroachment (Spicer et al., 2019) & field emergence (Hehenberger et al., 2019)	Field-level	Field emergence legitimizes new organizational form and functions	Encroachment, displacement, and suppression (used by those with power)
Scaffolding (Mair, Wolf, & Seelos, 2016)	Organizational	Form follows function (concealed goals pursued until altered institutional arrangements emerge)	Conceal goals (as a mechanism of power)
Systems hijacking	Organizational	Form follows function	Power by appropriating existing forms and working within prevailing system

First, systems hijacking takes the organization as the unit of analysis to understand how alternative organizations and their activities can affect and transform their institutional context. In contrast, most explanations of transformation focus on a constellation of mechanisms enacted by multiple actors within and across different sectors (Seelos & Mair, 2018). For example, institutional entrepreneurship literature emphasizes that it takes more than a single individual or organization to achieve transformation (Hardy & Maguire, 2008), and scholarship on organizational fields similarly describes emergence and change as a process enacted by multiple actors operating within and across organizations (Hehenberger et al., 2019; Spicer et al., 2019). Institutional conversion also envisions a coalition of political actors intervening in political spaces (Streeck & Thelen, 2005; Thelen, 2004). In the social movement scholarship, André Gorz’s (1967) and Nancy Fraser’s (2020) accounts of institutional transformation—both of which have a substantive influence on contemporary systems change theories—involve multiple actors engaged in social and political movements working across organizational boundaries. Our focus on the organization as the unit of analysis provides quasi-closure conditions and has analytical advantages for understanding causal mechanisms of observable change (Seelos & Mair, 2014). Systems hijacking belongs to this genus of systems change archetypes that seek to identify specific organizational activities that cause observable outcomes.

Second, our concept of systems hijacking seeks to explain how organizations work to change the function of existing capitalist forms and institutional arrangements in which they are embedded, similar to the process of institutional conversion. That is, the goal of the organizational actor engaged in systems hijacking is to re-purpose existing market-based forms and the systems in which these forms are embedded to serve new purpose (i.e., community and social goals). Contrast this with theories that emphasize change in the forms underpinning incumbent systems as the central goal, with the assumption that function will follow form. For example, Fraser’s (2020) solution to the recognition-redistribution dilemma requires remaking institutional forms along socialist lines to address economic and cultural inequalities. Her proposed resolution requires treating end-state outcomes as subordinate to the processes and institutions that produced them. Similarly, Gorz’s (1967) non-reform reforms emphasize transformation in institutional forms (e.g., degrowth policies), where it is implied that function will follow a change in form.

Although there is a risk that a focus on changing the function rather than form underpinning capitalism may “reinforce contemporary capitalism rather than heal capitalism’s social pathologies” (Mair & Rathert, 2021, p. 827), this focus on function has analytical advantages by evaluating success based on outcomes achieved. In other words, “complex systems change all the time in a dynamic manner without our interventions. Therefore, change per se is neither interesting nor difficult to achieve” (Seelos & Mair, 2018, p. 41). The idea that we can know what changes in the form of the system architecture underpinning capitalism will lead to desired outcomes assumes an enlightened predictive power of the new system’s architects. A focus on function over form also forces scholars to engage with unintended consequences of organizational interventions, an area that has been largely neglected in alternative organization literature (Mair & Rathert, 2021).

A third distinguishing feature of systems hijacking is the centrality of power to the mechanisms that alternative organizations use to affect and transform institutional arrangements underpinning complex social problems. Systems hijacking seeks to explain how the disenfranchised might compete in an institutional context that favors private interests by working within the capitalist system to use the same structures and systems in which they are embedded for new purposes. Existing explanations of transformation often understand power as something that must be dismantled by the disenfranchised rather than appropriated by them. For example, Fraser’s (2020) emphasis on redistributive policies is aimed at dismantling the root cause of inequalities rather than using them for new purposes. Similarly, while Gorz’s (1967) non-reform reforms envision a progressive appropriation of power by the disenfranchised, this is achieved by working against the prevailing capitalist system rather than within it.^2^ Institutional conversion has been applied to understand how disenfranchised groups might leverage existing institutions to achieve new purposes (Hacker et al., 2015), but how these groups first gain access to power or the mechanisms by which they achieve their goals are unexplained. Indeed, the relative power of the elite, as well as their interests in (Giridharadas, 2019), and ability to preserve, institutional arrangements that benefit them by encroaching on other fields (Spicer et al., 2019) and suppressing alternatives as new fields emerge (Hehenberger et al., 2019) is often missing from accounts of how such transformation could be achieved in practice.

To explore how alternative organizations could leverage existing forms and the power they afford their users, this article focuses on the commercial trust and the institutional arrangements that assign users of this legal form certain privileges. Tax sheltering, risk pooling, and transferability properties of these structures have allowed private wealth holders to invest and grow their wealth to vast sums in the Anglo American legal system (Langbein, 1997; Pistor, 2019). The theoretical motivation underpinning this article is to understand how alternative organizations could hijack these structures and the legal privileges that are assigned to them to build wealth for communities, and the consequences of leveraging inherited structures of capitalism for new purposes.

## The Great Wealth Transfer

The context for this article is one of the greatest transfers of wealth in global retail history. Local “main street” businesses are a critical part of community infrastructure and are being lost at an alarming rate across North America and globally. For example, in 2018, it was estimated that more than 700,000 Canadian small businesses were at risk of closing within 10 years due to lack of a succession plan (Geobey & Ronson, 2018), and similar trends are observed in the United States, with more than half of all privately held businesses owned by people above the age of 55 years, the majority of which do not have succession plans (Project Equity, 2020). The consequences of the pandemic—physical distancing requirements and increased consumer use of digital shopping, particularly among millennials and high-income earners (McKinsey, 2020)—are accelerating this hollowing out of local businesses from communities (Project Equity, 2020). If past crises are a guide for the future, the prolonged low interest environment intended to stimulate economic recovery could further exacerbate threats to small businesses by making it easier for larger investors to access cheap capital and buy up attractive local businesses and other assets with strategic value to communities (Baines & Hager, 2021). There is some evidence that this trend is already underway; it is estimated that the number of small business owners in the United States dropped from 15 million to 11.7 million in April 2020 (Fairlie, 2020).

Although the pandemic is exacerbating challenges facing small businesses and accelerating succession timelines, it is important to recognize the deeper structural and systemic issues in the market that have made main street businesses particularly vulnerable. Demographic aging means that retiring business owners have limited options to find willing local buyers (Project Equity, 2020), and small businesses have long faced asymmetric competition with larger corporations (Baines & Hager, 2021). For example, “even though locally owned businesses constitute slightly more than half of the US economy they receive far less than half of all banking capital and almost none of the securities capital tied up in pension funds, mutual funds and insurance funds” (Schumann, 2020, p. 26).

To be sure, initiatives exist to level the playing field, such as programs to increase small businesses’ access to government procurement and financing. But inequalities are rooted in a system that privileges the corporate form and private actors that have access to the legal system (Pistor, 2019). Successful small business owners who are unable to find qualified and interested local buyers face the hard choice of closing or selling—often at a discount—to large and distant buyers. For example, many successful Canadian small businesses are being sold to either American private equity firms or corporate competitors (Social Capital Partners [SCP], 2020). Community economic development research tells us this exodus of small business ownership and industry consolidation drains wealth from communities, with significant and detrimental impacts on local employment, local control of assets, and provision of critical goods and services, particularly in rural communities (Steiner & Teasdale, 2019). Given the magnitude of the small business succession cliff facing communities across North America (Project Equity, 2020), community economic development and policymakers are increasingly calling for alternative ownership models to keep these wealth-generating assets in communities (e.g., democracy collaborative).

### Existing Models for Alternative Ownership

While receiving limited scholarly attention, the practice of alternative organizations advancing approaches and solutions to complex social problems within market-based systems is not new. Alternative organizations have extensive experience leveraging existing market structures for social and community purposes and advocating for legal innovations that allow for alternative forms of ownership and control of businesses and other assets with strategic value to communities (Dubb, 2016; Spicer, 2020).

This article explores the feasibility of a community trust that could acquire multiple businesses, attract finance at scale to grow the businesses, and redistribute profits back to community. The model is guided by three key criteria that are necessary to compete with private interests for businesses with wealth-generating properties to keep wealth in communities. First, the model must be scalable to attract institutional investment and to replicate strategies of private investors. Second, the model must provide for durable ownership to prevent the future sale of businesses to private interests. Third, the model requires efficient representation of the plurality of community interests to make decisions at a pace that matches the market and inspires confidence among third-party investors. This section reviews prominent alternative ownership models in the Anglo American context to show a lack of models that are intentionally designed to meet all three criteria (see Table 2). To be sure, this is not a comprehensive review of existing community wealth-building models. Nonetheless, it provides a useful framework to assess alternative ownership models for our purposes.

**Table 2.** Review of Existing Community Wealth-Building Models.

Existing community wealth-building models	Scalability: model is used to hold ownership stake in multiple businesses while attracting additional third-party investment	Durability: model is used to hold assets in perpetuity and prevent sale to future owner that could change purpose	Efficient community representation: model is used to allow interests of multiple stakeholders from community to be represented efficiently
Community Development Finance Institution	X		
Steward-ownership trust (noncharitable purpose trust)		X	X
Employee-ownership trust		X	X
Co-operative with homogenous members (e.g., employee owned or producer owned)			
Multi-stakeholder co-operative		X	
Foundation-owned firm		X	X
Community Development Corporation		X	X
Investment **c**o-operative or social venture fund	X		X
Charitable Purpose Trust		X	
Benefit Corporation	X*		X
Community Interest Corporation		X	X

* If Benefit Corporation is an investment fund, for example, then scale is relevant.

### Scalability

A key condition for a model that has the potential to compete with private investors for business assets that have wealth-generating properties is scalability. Operating at scale would allow the community entity to attract third-party investment, including from larger institutional investors who often cannot deploy smaller amounts of capital due to transaction costs. Scale would also allow the entity to engage in value-generating strategies by holding ownership in multiple businesses. For example, the entity could replicate strategies used by private equity investors to generate value, such as by encouraging portfolio companies to work together, linking previously unrelated goods and services across industries to help companies unlock new sources of value for each other (Khairallah & Mann Quirici, 2021).

Although existing models, such as community development finance institutions (CDFIs) and investment co-operatives, allow communities to invest in multiple businesses, these models are not designed to compete with private equity investors to acquire a portfolio of businesses, nor are they designed to facilitate impactful exits that allow business owners to preserve community and social benefits. For example, CDFIs typically provide below-market capital to underrepresented entrepreneurs to start up their business or to fund growth and expansion of existing businesses in underserved communities (see Pinsky, 2001).

To be sure, some existing models could serve the function of providing alternative exit options for business owners seeking to preserve social purpose or community benefit of their business. For example, foundation-owned firms allow community to own business and attract third-party investment, but they are typically applied to holding a single firm, and there are limitations in using these legal forms in the North American context (Hansmann & Thomsen, 2021). In addition, steward and employee ownership trusts in the United States, and to a limited extent in Canada, provide business owners with impactful exit options (see Gary, 2019; Hansmann & Thomsen, 2021). Although these models could hold multiple business assets in trust, they typically hold a single firm.

### Durability of Ownership and Mission

A second key criterion for a community wealth-building model is durability of ownership. Existing corporate structures fail to offer protection of a company’s mission in perpetuity because future owners can change the structure. For example, incorporating as a Benefit Corporation to pursue nonfinancial goals alongside business purposes does not provide protection from future owners changing the structure back to the traditional corporate form (Gary, 2019). This possibility has also been observed among co-operatives; in 2020, an iconic Canadian co-operative—Mountain Equipment Co-operative—was sold to an American private equity investor.

In response to the challenge of durability, innovations in legal forms are being introduced both in North America and abroad.^3^ For example, noncharitable purpose trusts (steward-ownership trusts and employee ownership trusts)—where legally permitted—can lock in the purposes the founders have for the business. The trust arrangement allows community members, as beneficiaries of the trust, to have indirect ownership, but they cannot change the structure of the business or sell it to private owners. The model also allows for the possibility of outside investors without selling ownership and control through the issuance of nonvoting shares (Gary, 2019). Although the steward-ownership trust model offers an innovative structure for holding community wealth-generating assets in perpetuity, they are not yet widely used or understood by the market. Similarly, though employee ownership trusts have grown in the United States, the costs of setting them up may limit their growth in Canada (Social Capital Partners [SCP], 2020), and they may not be suitable for smaller businesses (Rosen, 2009). Outside of North America, Community Interest Companies, which provide the ability to lock in a company’s assets for public benefit, have enjoyed widespread use in the United Kingdom (Haugh, 2020).

### Efficient Forms of Community Ownership

A third key criterion for competing with private investors for business assets is efficient representation of community interests. Community is defined for our purposes as an aggregation of people who share features of group life, such as norms, traditions, social conventions, and relationships (Rodríguez-Pose & Storper, 2006). At the same time, our definition of community is intended to reflect a plurality of interests within community (Haugh, 2021). There is an inherent tension in defining community in this way. On one hand, features of community life, such as trust and social capital, can enhance economic transactions (Rodríguez-Pose & Storper, 2006), and in our context, they can help identify acquisition targets and facilitate transition of ownership. On the other hand, the economic advantages of community might be outweighed by the real and perceived inefficiencies of community ownership that make third-party investors cautious and decision-making slow (Fung, 2015). For example, community development corporations and co-operatives allow for citizen participation in decision-making, although these models often involve compromise between their participatory governance and their social objectives (Mitzinneck & Besharov, 2019).^4^ Similar tensions have been observed in the context of community-owned enterprises (Haugh, 2021).

The steward-ownership trust has the potential to resolve this tension by separating ownership and control. This separation makes community stakeholders—employees, suppliers, among others—indirect (beneficial) owners (Gary, 2019). Scholars suggest that the trust, as an alternative to the corporation, has advantages for aggregating community interests and the long-term preservation of social and community purpose (Gary, 2019; Tritt & Teschner, 2019).

In the Anglo American context, none of the existing models reviewed here fit all three criteria necessary to compete with private investors to acquire succession-ready businesses for community wealth building. To be sure, community wealth-building models in non-Anglo American contexts might offer some promising developments in this respect, but a review of these models is beyond the scope of this article. Although steward-ownership trusts offer durability and an efficient model for community representation, the model has not been used in ways that would allow for competing with private equity investors by managing a portfolio of businesses and attracting institutional investment. As Dubb (2016, p. 10) concludes in his review of community wealth-building models, “they certainly build wealth for their members, but they may fall short of becoming instruments of social transformation.”

## Method and Approach

Organizational scholarship is dominated by empirical accounts using historical data, leaving the emergence and potential of new processes and structures for re-imagining capitalism largely unexplored (Kornberger & Mantere, 2020). Indeed, as the analysis in the section above suggests, there are limits to relying on empirical data to assess the performance of alternative organizations in addressing complex environmental and social issues and transforming institutional arrangements underpinning capitalism. In many cases, it is too early to observe the impact of innovative organizational structures on transforming their institutional context. In other cases, alternative organizations (e.g., worker-owned co-operatives) have existed for many years but historically reflect a retrenchment from the market rather than a strategic attempt to compete with private investors for ownership and control over community wealth-generating assets (see Spicer, 2020).

In other disciplines, such as law and economics, thought experiments are extensively used to identify new areas of inquiry that are critical of taken-for-granted assumptions and that offer new insights and imagine new possibilities (Kornberger & Mantere, 2020). Take, for example, the planetary trust: Edith Brown Weiss’ (1983) thought experiment uses an analogy of the trust to imagine a scenario whereby current generations act as both beneficiaries of their ancestors and trustees for future generations in protecting environmental assets. In her thought experiment, no new information is introduced; analogical reasoning is used to generate new insights about achieving intergenerational equity. Brown Weiss’ thought experiment is more than “an argument dressed up in heuristically appealing clothing” (Gendler, 2000, p. 33); the experiment allows for other scholars (Clark & Woods, 2012) to introduce their own analogies and explore limits of the thought experiment in a rigorous way to further test and refute the hypotheses advanced by the experiment.

This article uses a thought experiment to imagine a community trust that could acquire and operate businesses with strategic value to their communities, attract investment to scale the businesses, and redistribute profits back to communities. The goal of the thought experiment is to explore the potential and limits of an alternative investment organization for building community wealth by constructing an internally consistent model within a possible world to confirm whether the imagined situation is feasible (Cooper, 2005). For example, feasibility is assessed by focusing attention on desirable outcomes (i.e., what ifs?) as the starting point of the analysis and working backwards to understand the unintended negative outcomes that would need to be disabled or suppressed by the organization to achieve them (see Seelos & Mair, 2014). In these ways, the thought experiment serves a creative function by helping generate, demonstrate, and communicate new ideas by asking a series of what if questions to facilitate this imagining from a familiar analogy to an unfamiliar context (Kornberger & Mantere, 2020).

The thought experiment also serves a second, critical, function by helping problematize existing theories about alternative organizing and community wealth building by showing their limitations, unintended consequences, and taken-for-granted assumptions (Kornberger & Mantere, 2020). In this way, “the thought experiment serves as an act of introspection that results in developing novel, justified beliefs about the empirical world and to bring to light unarticulated and implausible tacit beliefs” (Gendler, 2000, p. 411).

To be sure, there are limitations in this approach for developing insights that inform organizational design and decision-making. Thought experiments are not a substitute for empirical work (Kornberger & Mantere, 2020). For example, thought experiments cannot tell us how something worked or why. A second limitation of the thought experiment as a method relates to evaluating quality and validity (Gendler, 2000). Because the thought experiment occurs in the researcher’s mind, the experimenter must commit themselves to rigorously considering all relevant consequences in answering the what if questions (Cooper, 2005; Ketokivi et al., 2017). In addition to the completeness of the scenario’s exploration, thought experiments can be evaluated by the relevance of their core analogy and the generative potential of the structural connectedness between the analogy and problem setting (Ketokivi et al., 2017). The following section aims to provide a detailed account of the choice of the analogical source and the imagined scenario to allow for critical engagement with the thought experiment and generate relevant hypotheses that could be tested empirically in future research.

## What if . . .? Community Acquisitions at Scale

### Understanding the Problem

Imagine a rural community in North America that has undergone significant transitions typical of many towns with a high dependence on a single industry. Twenty years ago, the local mill cut its shifts by 60%, resulting in significant loss of local employment. Revitalization efforts successfully pivoted the town to a tourist destination with enough local jobs at the mill to sustain the population. But in recent years, retailers are struggling to maintain sales with increased digitization of the economy and large retail chains moving in south of town. The COVID-19 pandemic has amplified these challenges. For example, larger chains could remain open because they sell essential goods in addition to the wide range of other retail goods, while smaller and locally owned businesses were forced to close their doors.

To be sure, the pandemic has also elicited a strong demonstration of community support as residents pledged to shop local and support their communities: but there are only so many pottery dishes, chocolates, and bicycles that residents can purchase. Without capabilities to extend into other markets, main street businesses face limited growth opportunities. Although some rural communities are experiencing a population boom because of being priced out of real estate in urban centers and the flexibility of working remotely in the pandemic context, this is not translating into new business start-ups in these regions.

Government officials and well-intentioned philanthropists meet to discuss their plan for economic recovery and renewal. Infused with a belief in the power of market-based reforms and entrepreneurship for solving social problems, the plan is to attract private investment to make tractable progress on social issues at scale and overcome many of the challenges with traditional philanthropy and government funding that relate to a lack of accountability for outcomes and dependency issues, among others.

As customary in thought experiments in organization studies (Simon, 1991), an alien drops into the meeting. At first, the alien understands the logic of what is being proposed; private capital encourages innovation and accountability for outcomes that transcend challenges with conventional philanthropy and public granting programs. But then, the alien begins to wonder how the proposals will affect those that the plan is intended to benefit. To be sure, private investment, combined with venture philanthropy, could stimulate new economic activity. But who gains from these investments? And while the idea of encouraging nonprofits in the community to become more entrepreneurial by starting social enterprises is enticing, in absence of broader investments and structural reforms, will not these social enterprises suffer the same fate as small businesses in the community? Social enterprise may be even more vulnerable given the added burden of balancing social with financial goals. Another concern is the question of who makes decisions about where this capital would be deployed. Although some investors would be local, others have little knowledge of what the community needs.

The alien also observes that there is no incentive to change the systems in which wealth is accumulated and, as such, power remains with investors and funders to decide what assets are worth investing in and saving for the community. To be sure, some participants recognize the need to shift power to community. These enlightened participants are self-reflexive of their role in perpetuating the inequalities they claim to be concerned with solving and are seeking to use their power and investments to address these structural and systemic challenges. But their efforts are overshadowed by inertia of a system that makes communities dependent on corporations and investors donating or investing a fraction of wealth they have extracted from communities through their own entrepreneurial and investment activities back into communities.

### The Analogy of a Commercial Trust

The commercial trust (see Tritt & Teschner, 2019) provides a basis for imagining a community wealth-building scenario that responds to the challenges of small business succession and hollowing out of community assets, while also engaging with questions of power. This section begins with a brief discussion of the commercial trust to understand its historical context, structure, and core functions. The section then demonstrates the suitability of the commercial trust as the analogical source for community wealth building through a series of what if questions designed to explore the potential and limits of the model in this new context.

#### Commercial trust: historical context, form, and function

A trust is a legal arrangement between three parties: a settlor, a beneficiary, and a trustee. The legal scholarship on trusts has, until only recently, focused on testament trusts: trusts that facilitate the gratuitous transfer of wealth from a wealth holder (settlor) to their family members (the beneficiary), where the trustee is usually a close personal contact such as a family member or neighbor (Langbein, 1997). The primary function of a testament trust is to transfer wealth in ways that limit tax liabilities and to assign control over assets intended for a beneficiary to a responsible party until the beneficiary reaches an age where they are capable of managing the assets themselves. Charitable trusts also govern donative transfers for the benefit of purposes that are legally defined as charitable.

Significantly less attention has been dedicated to the commercial trust, defined for our purposes as a legal entity that is separate from its three principal parties—trustees, beneficiaries, and settlors—and provides its users the potential to insulate assets from personal creditors much in the same way that we understand corporations to be governed by organizational law and not contract law (Hansmann & Mattei, 1998).^5^ As some scholars point out, this neglect of the commercial trust is surprising given the pervasiveness of the commercial trust arrangement that underpins pension funds, mutual funds, and a range of other investment organizations (Langbein, 1997; Tritt & Teschner, 2019).

In the commercial context, the typical wealth holder, instead of transferring property for their surviving family, is an investor buying shares in an asset pool for the investor’s own benefit (Pistor, 2019; Tritt & Teschner, 2019). In this way, the settlor of the trust is often the same as the beneficiary. Trustees of commercial trusts are often professional service providers that charge a fee to manage a complex pool of assets on behalf of the settlor-beneficiaries. Commercial trusts allow investors to pool risk and securitize assets. Scholars have documented how these investment organizations have allowed third-party investors, as beneficiaries of the trust, to amass large sums of wealth through a tax efficient structure (Langbein, 1997; Pistor, 2019).

The following thought experiment considers the potential of alternative organizations to leverage this same legal form—the commercial trust—for building community wealth by acquiring and controlling businesses with wealth-generating potential and attracting additional investment to grow and scale the acquired businesses.

### What if Owners Could Donate or Sell Their Business to Community?

Some small business owners who do not have a successor or who have built substantive wealth outside of their business express interest in donating their business to their community. These owners are often motivated by both the desire to leave a legacy and the tax benefits associated with such donations. Indeed, capital gains tax associated with selling shares of a business can place a significant burden on small business owners (Bove, 2004). The business owner may also want to avoid a sale to a nonlocal buyer and maintain local jobs in the community or the owner may be a nonprofit entity operating a social enterprise and want to ensure that the business remains in service of community.

The current option available to these business owners is to seek out a charitable community trust or foundation that has the skills and capacity to accept a business as a donation. Although this option addresses a seller’s motivation to avoid capital gains tax (at least in the United States—Canada does not incentivize the donation of private shares or real estate at the time of writing) and give back to their community, it does not guarantee the continued legacy of the business in community. This is because charities laws in Anglo American jurisdictions restrict foundations and charitable trusts from continuing to operate the business (Hansmann & Thomsen, 2021). In the United States, recent changes to charitable trust law have allowed some leeway for private foundations. But in general, operating a business that falls outside of the related business activities of the charity and owning majority shares of a business would be considered an active investment and, therefore, not permissible under tax laws.

As such, foundations and charities with the capacity to accept a business as a donation will, in most cases, be required to dispose of its shares or assets. For those community foundations and charities that do not have the capacity to accept a business as a donation, a business owner would be required to sell the shares and donate the cash within a specific time period to avoid capital gains tax. But many small business sales depend on vendor financing arrangements that provide for buyers to pay the total funds of the sale over multiple years. As such, sellers often do not have the money from the sale of their business available to donate within a limited time frame (e.g., 30 days) and will not be able to take advantage of the tax incentive program. For those that can liquidate shares and assets of the business, the money is pooled with the foundation’s other assets and invested in capital markets to generate financial returns that can, in turn, be used to support local community initiatives through grants and donations to qualified donees. Often, these investments are made in conventional assets and do not benefit local communities.

Moreover, there is no guarantee that the business will be sold to a local buyer to maintain jobs and other benefits associated with having the business anchored in the community. For businesses with a social purpose (i.e., social enterprise), there is no legal mechanism that allows for ensuring the new owner maintains a commitment to this purpose (see Gary, 2019). There is significant risk of the social value of the business for local community being lost through its sale in the open market. Moreover, the costs and fees associated with arranging the sale detract from the overall value of the donated business.

### What if Communities Could Leverage the Corporate Trust to Acquire Business and Operate Them for the Benefit of Community?

In our scenario, the commercial trust serves as an analogy for a structure that could allow the nonprofit and community sector to accept privately owned businesses as donations or purchase businesses through an open market transaction or combination of donation and sale and then operate the business arms-length in a way that continues to benefit community. In some cases, there may be opportunities to enhance benefits to community, such as by growing and scaling the business to serve new market segments, including underserved populations, and providing new employment opportunities to persons facing barriers to employment.

An example of this model is the university trust. Universities in North America have used the commercial trust structure to build wealth and generate revenues that are then used to support the university’s charitable mission. In this model, the university is both the settlor and the beneficiary. As the settlor, the university entrusts assets—usually land—to a trustee that is set up as a separate incorporated legal entity. The trustee is responsible for securing finances and developing the land into a wealth-building asset. The university is typically the sole shareholder and, as such, is also the beneficiary of the trust. As a beneficiary with charitable status, the university is exempt from taxes on earnings from the investment trust, provided the assets are transferred from the trust to the beneficiary on a regular basis in accordance with tax requirements. Although real estate development has been widely used in the university trust model, university trusts have invested in other asset classes, including private equity investments.

What if communities could use this commercial trust structure to acquire and operate small business and other assets that are core to the community and manage those assets in perpetuity for the benefit of community? A separate legal entity created with a trust relationship to community could acquire and manage small businesses and professionalize asset management. The entity would have the legal authority to engage in commercial activities, operating at arms-length from beneficiaries. All profits generated would be re-invested in the businesses or donated back to the community. In this scenario, small business owners that do not have qualified and interested local buyers and who meet specified criteria could donate or sell their business to the trust and keep their business and its associated benefits in the local economy. The trust would assume ownership and operate the businesses for the benefit of community. The trustee, as a separate incorporated entity, would bring professional management expertise required to strengthen the business model and attract additional finances to grow and scale the businesses.

The trustees would also be tasked with converting acquired businesses to a social purpose. As such, the selection criteria for determining which businesses to acquire are critical. Businesses that depend on strategies or models that are fundamentally inconsistent with the community purpose of the trust would be screened out. Businesses that offer good growth potential but that lack strong connection to community could also pose challenges to trustees seeking to balance profitability with community benefits.

From the community perspective, a centralized commercial trust to acquire local businesses raises questions around what counts as local ownership. A single, rural community may be too small to operate the trust and economies of scale are necessary to ensure the financial sustainability of the model. Indeed, a commercial trust could be perceived as an outside and distant investor. That is, there is a risk that the community trust starts to resemble the prevailing model of distant ownership and control. As one cautionary tale, regional policies in the United States to address systemic economic inequalities have widely failed, and analysis has surfaced deep structural problems with intended solutions offered at regional levels that suppress recognition of sub-regional differences and treat regions as static concepts (see Moore, 2005). Optimal scale of the trust and how community is defined and represented are key considerations in determining the governance of the trust.

### What if the Trust has Multiple Beneficiaries With Diverse Interests?

With few exceptions, trust law requires a clearly defined beneficiary that can enforce the trust to be considered legal. In our example above, the university is the beneficiary. As a clearly defined legal entity, the university could enforce the trust if the trustees failed to manage the portfolio of assets in the university’s best interests. In our scenario, community is the intended beneficiary. But community is an ambiguously defined beneficiary, which means the trust—if structured as a purpose trust—would likely fall into the category of a noncharitable purpose trust. Until recently (in some jurisdictions such as Oregon), noncharitable purpose trusts have not been recognized by the courts as legitimate (see Gary, 2019), and in many jurisdictions, they remain illegitimate forms. Although innovations such as the perpetual purpose trust legislation introduced in the State of Oregon provides for multiple and undefined beneficiaries of a noncharitable purpose trust (see Gary, 2019), as discussed above, this model is typically applied to ownership of a single firm and does not address our scalability criteria.

An alternative way to give legal effect to the definition of community would be to assign claims to distinct beneficiaries in the same way that the commercial trust allows for multiple beneficiaries. Community members—local business owners, employees, nonprofits, and charities, among others—could be allocated residual rights over the trust assets through the purchase of trust certificates. In this way, multiple community members can become both the settlors and beneficiaries of the trust. The interests of settlors and beneficiaries would be aligned, as both seek preservation of value. For example, as both the settlors and beneficiaries of the trust, employees want to see businesses continue to operate in their communities so they can remain employed. Similarly, nonprofits and charities may be interested in earning a modest return that is comparable with what they receive on their existing investments but would be primarily interested in the opportunity to invest in local community. Small business owners want to see their legacy in community preserved. None of the beneficiaries have an interest in excessive risk-taking.

However, there is potential for conflict arising among the different groups of settlor-beneficiaries. Indeed, such conflicts have been observed in conventional commercial trust settings, raising questions around how the beneficiary interest is defined in the trust relationship. In a commercial trust, the residual owners (settlors/beneficiaries) are aligned in their interest of generating a financial return on their investments. But scholars have documented several cases where the interests of some beneficiaries conflict with the interests of others. In the pension fund trust, for example, scholars have documented the conflict of interests between current and future generations (Clark & Monk, 2017).

In our scenario, these tensions are exacerbated by the diversity of beneficiary interests in the trust. That is, unlike the pension trust, where beneficiaries share common traits of being employees and eventually retirees, in our scenario, community is represented by a diverse range of beneficiaries. These tensions have been well rehearsed in the social enterprise literature. For example, employees of community-owned businesses benefit from living wage policies, but this could reduce the profits that support nonprofits and others, particularly in sectors where the market conditions do not support living wage. Efforts to hire persons facing barriers in the business portfolios could also undercut productivity of a business and its potential to grow, if not effectively implemented.

As such, the tensions that social enterprises and other social purpose businesses must manage with respect to mission and profit are not resolved through the trust model but are shifted from individual social enterprises to the commercial trust entity to manage. The difference is that under the commercial trust model, these tensions are governed under fiduciary duties. Fiduciary duty requires impartiality in their treatment of beneficiaries and imposes a duty of loyalty and prudence that requires trustees to act in the best interest of beneficiaries (Tritt & Teschner, 2019).

We can imagine some resolutions to these tensions between beneficiary interests afforded by the duty of impartiality interpreted in statutes governing fiduciary duty. For example, if the threshold for assessing community benefit is that the business remains locally owned and local ownership serves as a proxy for other benefits to communities such as local employment and contribution to the tax base, trustees could be required to manage business in a way that is consistent with shared value such that ancillary goals related to increasing wages for employees or pursuing other community benefits, so long as they contribute to the profitability goals of business.

But we are placing faith in local and community ownership as a proxy for positive societal and community impact. As one community sector leader engaged in discussion to help the researcher develop initial ideas for the thought experiment points out, “even small and locally owned businesses can exhibit the worst of capitalism where profits are ruthlessly pursued at the expense of the community” (personal communication, November 15, 2020). It follows that the use of a commercial trust structure is not sufficient to ensure that community benefits are protected from private interests of community members. Elsewhere, scholars have argued that using inherited forms pose significant challenges for innovation of investment practice (Clark & Monk, 2017), and governance is key to transcending barriers to innovation in the context of exercising sustainable investment mandates (Clark & Monk, 2017; Clark & Urwin, 2008).

To understand how governance can be effectively designed to transcend barriers to innovation in investment management that allows trustees to balance community and financial objectives, it is useful to consider the advantages of the trust over the corporate form to achieve the objectives of holding and managing multiple businesses for community (e.g., a holding corporation). Unlike the corporate form, the trust allows for flexibility in drafting governance documents that could be written to require trustees to pursue community benefit alongside financial returns (Tritt & Teschner, 2019). Provided the governance instrument is effectively drafted, beneficial owners of a commercial trust formed with the purpose of achieving community benefit could not sue a trustee for breach of its fiduciary duties because the trustee was pursuing a management model that seeks to achieve both community and financial objectives (ibid, 2019). This is because trustees are not directly accountable to residual claimholders but, rather, to the governing instrument of the trust. The trust also has distinct advantages over the corporate form in its flexibility to adapt its governance instruments over time (ibid, 2019). This would allow the trust to adapt to changes in market conditions and community interests that require new interpretations of the balance between community benefit and financial goals.

### What if the Settlor-Beneficiaries Want to Attract Additional Capital to Scale?

The sale of residual claims (trust certificates) to community members would provide the trust with modest funds for acquiring businesses. However, to invest in the businesses and grow and scale their benefits to community, additional capital is required. This capital could be raised through the sale of residual claims to third-party investors. Indeed, in our thought experiment, we have thus far neglected one of the most attractive features of the commercial trust: the securitization of assets and pooling of risk.

Access to finance is a significant challenge for social enterprises and small business owners; investors often struggle to find social impact deals that align with their risk-adjusted return objectives and deal size. From the investor perspective, a trust that acquires and manages multiple social enterprises in a professional manner provides an attractive impact investment opportunity that addresses these challenges. Securitization separates the assets into a trust that is distinct from the rest of the liabilities of the settlors. Because these assets are specialized, they are easier for outside investors to evaluate than evaluating individual nonprofits and their capacity to operate a successful social enterprise. The trust would also offer investors scale and securitization that protect investors from risks and assurances of professional management and due diligence. From the perspective of the community sector, the model offers access to institutional investment that otherwise would not reach the sector due to the challenges noted above. The trust could also enter into deals as the limited partner on projects that are of value to the community.

Although the upsides of using the model to attract third-party investment are obvious for both investors (addresses scale and due diligence challenges) and the community sector (addresses access to finance challenges), we must also consider the potential negative implications of inviting third-party investment. The sale of residual claims to third-party investors would create potential for conflict between settlors and some of the new beneficiaries (third-party investors). This is because risk preferences differ now between some of the beneficiaries and the settlors. In contrast to our previous scenario, where beneficiaries and settlors of the trust were the same entities, and both shared interest in preservation of capital, our new beneficiaries (third-party investors) have interests that closely reflect shareholders of the corporate form seeking risk-adjusted returns. These residual claimants may want to see trustees take higher risks than the settlors of the trust (employees, nonprofits, etc.) would prefer.

The consequence of inviting third-party residual investors is that it mutes the benefits of fiduciary governance. This is because the commercial trust begins to face pressures to be governed more like a corporation, where shareholders demand power in exchange for their capital at risk (see Schwarcz, 2003).

Some investors may be willing to accept capped returns although the potential for attracting investment at scale that is a distinct advantage of this model may require targeting market-rate returns. And while philanthropic or government entities could provide concessionary capital to attract mainstream investors to the model, this raises new questions around the value of subsidizing investor returns that have long been raised as a criticism of social finance and impact investing (Edwards, 2007). At best, we are left with a model that strives to replicate the approach that private interests have taken to build wealth through leveraging the commercial trust once we invite third-party investors to achieve scaling goals.

Without caps on investment returns and governance that restricts the influence of third-party investors, extending the model to outside investors brings us closer to the conventional corporate form, where investors have control through directors to extract financial returns. As such, there is a premium on governance to ensure that trustees have the authority to pursue social impact and community benefit goals alongside financial goals and to do so in a way that protects the long-term success of the business’ underlying social purpose and value-maximization for all stakeholders.

## Discussion

### Theoretical Contributions

The intention of the thought experiment is to explore the feasibility of a new model for building and maintaining community wealth through acquiring succession-ready local businesses at scale and managing them through a trust entity. The thought experiment was used to critically reflect on the prevailing models of community ownership of businesses that fall short of transformational change and to imagine how alternative organizations could leverage the commercial trust structure through a series of scenarios to explore the conditions for success and limitations of the model. Our thought experiment set out to elucidate how the alternative organizations that engage in market-based activities can hijack existing structures and the institutional arrangements in which they are embedded to serve new purposes and, in doing so, transform the social systems that lock in community wealth erosion. The experiment adds to our understanding of how alternative organizations can affect and transform institutional arrangements and their limits by treating power as a central feature of alternative organizing.

There are, of course, alternatives to hijacking the organizational structures and institutional arrangements that private interests have leveraged over centuries to build and protect wealth. For example, some scholars have proposed the need for new legal forms that are fit-for-purpose, noting the challenges of using inherited or borrowed forms of capitalism for social or community purposes. Steward-ownership trusts represent a new organizational form in the U.S. context that is intended to address the succession planning challenges facing businesses with a social purpose by separating ownership and control for efficient representation of community interests, while also ensuring durability of business in community ownership (Gary, 2019). But the process of spreading innovations historically has been slow (Spicer, 2020), and there are several barriers to scaling alternative ownership models, such as the embedded agency paradox highlighted by institutional scholars (Hardy & Maguire, 2008) and the lack of understanding among the market and the institutional work required to build the ecosystem of support and community capacity to enable innovations (Spicer, 2020).

Our thought experiment highlights the possibility of leveraging inherited forms for new purposes to bring about transformation in social systems in the immediate term. But this approach has unintended consequences. There is risk in that using inherited forms embedded in our current capitalist system, power is shifted to other entities that claim to represent community, and ultimately creates new inequalities. Indeed, some scholars have concluded that using the same structures and systems underpinning capitalism will inevitably lead to replicating past systemic inequalities because “elevating new claims by bestowing on them legal protection of the kind that capital has enjoyed for centuries does not change the system; it reproduces it” (Pistor, 2019, p. 230).

This result is often presented as a dilemma: Working within the existing capitalist system often undermines the more transformative potential of engaging in institutional work to create new structures and systems that are aligned with social and public purpose (Schneider, 2020). But the long time horizon associated with institutional work to change dominant belief systems and ideas underpinning them is misaligned with the urgency of social and environmental challenges facing society. Framed as a dilemma, neither approach offers a feasible pathway to sustainable transformation in the institutional arrangements underpinning the problem of community wealth erosion; introducing innovations that aim at directly changing the form and systems in which they are embedded fails on a practical level and leveraging existing forms and systems in which they are embedded to serve new purpose fails on a substantive level.

The main finding from our thought experiment is that governance can transcend the dilemma associated with using inherited forms for new purposes. That is, there is a premium on good governance to manage tensions and balance representation of community with market-based discipline that is required to attract investment and scale to compete with private investors. Communities across North America face immediate threats from corporate concentration, which has been exacerbated in the pandemic context. They cannot afford to wait for legal innovations that allow for alternative ownership become widely accepted and used. Organizational governance serves as a key mechanism for holding tensions in balance to allow for the deeper institutional work (Lawrence & Suddaby, 2006; Tracey et al., 2011) that is needed to introduce new forms of organizing business and investment at scale.

In this way, different theories and levels of analysis (e.g., individual, organizational, field, institutional) are best viewed as complementary—and not competing—visions for achieving transformation in systems that lock in wealth inequality. Alternative organizations may initially engage in using the same structures and legal arrangements that have long provided private asset owners with power to maintain and grow their wealth. As these models are opened to third-party investment to scale and maintain competitiveness with private investors, the model will resemble many features of the traditional corporate form. Governance can sustain tensions in the immediate term until new forms are legitimized that are better able to deal with the complexities of community ownership. In absence of innovation in form, the power balance will revert to the current state, as private investors exert control over the entity.

### Limitations, Future Research, and Conclusion

Although thought experiments can help us understand problems with prevailing models and imagine new models and solutions, they cannot tell us about what is or what should be (Kornberger & Mantere, 2020). The significant empirical limitations of our approach provide a variety of important questions for future research. First, research is needed to empirically explore and further develop the concept of systems hijacking. Systems hijacking is presented in this article as a conceptual proposition. The article has not provided a detailed empirical account of the process and mechanisms by which the system that locks in the problem of community wealth erosion is changed by alternative organizations. Contrast this with the careful and detailed account of scaffolding as a process for changing systems of inequality through using empirical data collected over several years presented by Mair, Wolf, and Seelos (2016). To assess how systems hijacking works to address systemic inequalities, empirical accounts are needed to document the stages, processes, and mechanisms involved and to consider the consequences of this systems change archetype over time.

Second, and related to the previous limitation, we need a better understanding of the archetypes of transformation. There are many explanations of systems change and institutional transformation (Speth & Courrier, 2020, for review), but we do not have analytical mechanisms for comparing and evaluating their contributions to addressing contemporary challenges facing society, nor do we have a framework to understand the interactions across organizational level and other levels of action to change institutional forms and functions. There is value in pursuing more research to understand the role of alternative organizations in contributing to institutional transformation given the urgency and scale of challenges they face. This article has offered three criteria for comparing these archetypes: unit of analysis (e.g., organizational, field level, system level), theory of change (function vs. form), and the role of power. Future research could build on these criteria to make explicit the key assumptions underpinning theories of systems change and transformation, and to identify the organizational mechanisms, forms, and activities that enable different types of transformations, such as by providing an organizational-level mechanism to explain the process of institutional conversion. Thought experiments could help with this research agenda by suspending empirical constraints to allow space for imagining other pathways and scenarios for transformation.

At a time when economic and social inequality is demanding heightened attention and urgency in the context of the COVID-19 crisis, alternative investment organizations that seek social and community benefits alongside financial returns call for optimism as well as caution. Equally, as organizational scholars, we must be careful to avoid projecting our research agendas onto the pandemic context and to maintain focus on studying rather than co-performing transformation (Roth, 2021). To be sure, community wealth-building models offer a promising way forward in addressing inequalities by assigning ownership and control of wealth-building assets to communities. This goal could be achieved by leveraging the same structures that have allowed private interests to accumulate and protect wealth. But we need a better understanding of the conditions under which these alternative investment organizations can sustain their dual financial and social impact goals, as well as the potential for unintended negative consequences. Indeed, hijacking organizational structures and the systems in which they are embedded for new social or public purpose can accelerate progress toward addressing inequalities, but it can also import and disguise the same mechanisms that perpetuate the inequalities these models are intended to address. Organizational governance can play a critical role in navigating tensions and supporting conditions for innovation in investment management that is needed to respond to complex and rapidly changing environments.

Further developing research on alternative investment organizations will not only provide a better understanding of the conditions under which these organizations can absorb tensions social enterprises and other alternative business models face and manage them at scale but also contribute more broadly to research in alternative organization scholarship that has focused on social enterprises—and issues of their internal hybridity—while largely neglecting the form, function, and governance of the organizations that own and invest in these entities.

## Notes

1. Other mechanisms for systems change used by alternative organizations that scholars have highlighted include attracting a system to a parallel system with socially desirable properties and creating a subsystem within a system (Seelos & Mair, 2018).

2. Non-reformist reforms “aims by means of partial victories to shake the system’s equilibrium profoundly, to sharpen its contradictions, to intensify its crisis, and, by a succession of attacks and counterattacks, to raise the class struggle to a greater intensity, at a higher and higher level” (Gorz, p. 181).

3. Foundation-owned firms allow for ownership of businesses in perpetuity but, as noted above, legal context prevents their widespread use in North America.

4. For example, Dubb (2016) refers to employee stock ownership plans (ESOPs), which often lack employee representation on their boards, and consumer co-operatives, which often “emulate their corporate counterparts rather than see themselves as constructing economic alternatives.”

5. There is significant debate within literature on whether commercial trusts are legal entities in their own right, similar to corporations, or belong to contract law (as agreements rather than entities). For detailed discussion, see Tritt and Teschner (2019).
